# PD-1 Bispecific Killer Engager (PD-1 BiKE) effectively depletes effector T lymphocytes in experimental autoimmune encephalomyelitis

**DOI:** 10.3389/fimmu.2025.1644903

**Published:** 2025-08-13

**Authors:** Lauren C. Naatz, Shuyun Dong, Yujia Zhai, Brian Evavold, Mingnan Chen

**Affiliations:** ^1^ Department of Molecular Pharmaceutics, University of Utah, Salt Lake City, Utah, UT, United States; ^2^ Department of Pathology, University of Utah, Salt Lake City, Utah, UT, United States

**Keywords:** bispecific killer engager, autoimmune disease, experimental autoimmune encephalomyelitis, natural killer cell, PD-1 positive lymphocyte, effector T lymphocytes

## Abstract

**Background:**

Bispecific killer engagers (BiKEs), which harness natural killer cells to deplete target cells, have garnered success in ablating tumor cells but have not been well explored in eliminating primary cells, such as effector cells in autoimmune diseases. Previously, we reported a BiKE that targeted human lymphocytes expressing programmed death-1 (PD-1). The BiKE was shown to promote NK cell-mediated depletion of PD-1+ cells in vitro. Here, we posited that a mouse-specific PD-1 BiKE could be used as a tool to deplete PD-1^+^ cells *in vivo*.

**Methods:**

PD-1 BiKE was designed and produced in an IgG-like format. The BiKE was characterized for its functional binding, ability to facilitate NK cell-PD-1^+^ cell-cell interactions, and depletion of PD-1^+^ cells using several in vitro assays. The BiKE was then evaluated for its ability to deplete PD-1^+^ T cells *in vivo* using an EL4 tumor model, and the EAE model.

**Results:**

PD-1 BiKE demonstrated selective binding to PD-1^+^ T cells encompassing both a cell line (EL4) and primary cells. PD-1 BiKE simultaneously engaged its two targets, PD-1^+^ and NK cells, and mediated a 63% increase in cell-cell interactions between the two targets. In co-cultures of primary PD-1^+^ T cells and NK cells, the BiKE reduced the number of T cells by 28%. Importantly, PD-1 BiKE did not reduce PD-1^-^ T cells when co-cultured with NK cells. *In vivo*, PD-1 BiKE reduced the fraction of inoculated EL4 cells by ~53%. In EAE mice, PD-1 BiKE reduced the average number of primary PD-1^+^ T cells by 56% and 65% in the spinal cords and brains, respectively. Beyond the IgG-like BiKE, two non-IgG-like BiKEs were also designed and generated and demonstrated strong but distinct binding to PD-1 and CD16.

**Conclusions:**

The IgG-like PD-1 BiKE bound to both cellular targets, CD16 and PD-1, and was able to deplete primary PD-1^+^ T lymphocytes in the EAE model. Altogether, the work showcases the effectiveness of using BiKEs to deplete non-malignant cells.

## 1 Introduction

Bispecific killer engagers (BiKEs) have emerged as useful tools that leverage the cytotoxic mechanisms of natural killer (NK) cells to deplete target cells in cancer indications. (1) By engaging activating receptors on NK cells such as CD16 (FcyRIII), Nkp46, and NKG2D through scFv-derived binding moieties, BiKEs can simultaneously bind to a target cell antigen and activate the NK cell to kill the target cell (2). Importantly, BiKEs have shown superior efficacy in depleting target cells compared to conventional depleting antibodies (3–6). This is primarily due to the specificity, high affinity, and number of binding domains for activating NK cell receptors that can be engineered into BiKEs compared to the Fc of a conventional antibody, which is limited in all three aspects.

Apart from their mechanistic advantage, BiKEs also have design flexibility, making them adaptable to different clinical needs. There are several reported BiKE structures, broadly classified as either IgG-like or non-IgG-like formats. While IgG-like BiKEs typically resemble a native antibody and contain an Fc, non-IgG-like BiKEs are smaller and are devoid of an Fc (7). Reported advantages of an IgG-like BiKE in include longer serum half-life, increased stability, and improved solubility (8) (7), while non-IgG-like BiKEs may encompass a simpler production process, lower immunogenicity, and increased tissue penetration (7, 9), With advantages and versatility, the BiKE approach has attained tremendous progress in clinical translation and has shown safety and efficacy in several clinical trials (10–17).

BiKEs have been investigated primarily for their use in depleting malignant cells, in contrast, there has been little investigation of BiKEs in depleting non-malignant, primary cells, such as pathogenic lymphocytes found in autoimmune diseases. Meanwhile, depletion of lymphocyte subsets—such as those targeted by ocrelizumab (anti-CD20) or teplizumab (anti-CD3)—has proven effective in treating various autoimmune diseases. Thus, translating BiKE technology to deplete primary lymphocytes in the context of autoimmunity is both intriguing and promising. Though this translation of technology appears straightforward on the surface, it is embedded with many uncertainties and requires thorough investigation – particularly due to the innate differences between malignant cells and primary cells. Malignant cells often express mutated surface proteins, downregulate expression of MHC molecules, or overexpress death receptors, allowing for recognition and activation of NK cells (18); primary cells may not demonstrate these “red flags,” thus NK cells may not activate against them as readily (18–20). Further, while the target surface proteins of cancer cells are often constitutively and highly expressed (e.g., breast cancers express 2 million HER2 molecules per cell) (21), primary cells usually have moderate to low target expression (e.g., 2500 PD-1 molecules per T cell) (*unpublished data*). Additionally, the inflammatory environment in a given autoimmune disease may be different from a tumor environment, especially regarding the proximity of cytotoxic NK cells in the respective disease tissue sites (22, 23). Hence, broadening the use of the BiKE technology to autoimmunity still renders unknowns and opportunities.

Autoimmune disease treatments have advanced with the approval of new immunotherapies and the development of experimental approaches such as CAR-T cells (24–26). However, these therapies share two key limitations rooted in imprecise targeting. First, most therapies focus on a single pathogenic lymphocyte subset (e.g., depleting CD20^+^ B cells), even though multiple lymphocyte types contribute to autoimmune disease progression (27), thereby limiting their overall efficacy. Second, they often compromise naïve lymphocytes and their associated repertoires, leading to long-term lymphopenia and impaired immune regeneration (28–30). To overcome these challenges, we have explored a novel strategy targeting cells that express programmed death-1 (PD-1), which may improve both safety and efficacy. PD-1 is a key marker of effector T and B cells but is largely absent from naïve T and B cell populations (31, 32). Effector T and B cells interact and collaborate in driving autoimmune pathology (33–36). Therefore, depleting PD-1^+^ effector lymphocytes could provide a more comprehensive approach to halting autoimmune progression, while sparing healthy, naïve lymphocytes and preserving long-term immune competence. In fact, we have shown that the specific depletion of PD-1^+^ cells can reverse or delay autoimmune disease progression in animal models of multiple sclerosis (MS) and type-1 diabetes, without negatively impacting healthy immunity (37, 38). These proof-of-principle results were generated using immunotoxins, which have limitations as autoimmune disease therapeutics. Thus, to generate more clinically appealing therapeutic platforms, we employed the BiKE technology.

We produced a human-specific IgG-like BiKE targeting PD-1 and CD16 on NK cells (39). The BiKE was effective at eliminating PD-1^+^ cells *in vitro*, though it is unknown whether it could deplete the PD-1^+^ cells *in vivo.* To investigate whether the BiKE technology could be used to deplete the primary cells relevant to autoimmunity, we designed a mouse-specific IgG-like PD-1 BiKE and tested its efficacy *in vivo*. Altogether, this work demonstrates the translation of the BiKE technology for the depletion of primary lymphocytes.

## 2 Materials and methods

### 2.1 Cell lines and primary cell maintenance

EL4 cells (ATCC, TIB-39^®^) were maintained in DMEM supplemented with 10% HS; EL4 PD-1^KO^ cells were generated as previously described (37) and maintained in the same media in 5% CO_2_ at 37°C. Primary NK cells were isolated from mouse spleens using negative magnetic separation (STEMCELL Technologies, cat. 19855) and maintained in RPMI with 10% FBS, in 5% CO_2_ at 37°C. Primary CD4^+^ T cells were isolated from mouse spleens using negative magnetic selection (Miltenyi Biotech, cat. 130-104-454) and cultured in RMPI containing 10% FBS; for stimulated primary T cells, plates were coated with 1 ug/mL anti-CD3e (InVivoMab, cat. BE0001-1) and media was supplemented with 3 ug/mL anti-CD28 (InVivoMab, cat. BE0015-5); cells were maintained at 5% CO_2_ at 37°C. PBMCs were obtained from mouse spleens after disruption of tissue and straining through a 70 μM cell strainer.

### 2.2 Production of PD-1 BiKE constructs

Gene sequences for anti-PD-1 antibodies and anti-CD16 variable domains were obtained from published sources (40–42). For IgG-like PD-1 BiKE, the heavy and light chain gene sequences were cloned into separate *pcDNA3.1* mammalian expression vectors. For non-IgG-like BiKEs, each protein gene sequence was cloned into one respective *pcDNA3.1* expression vector. All plasmids were amplified in *Escherichia coli.* Plasmid DNA was purified using ZymoPURE II Plasmid Midiprep Kit (Zymogen, cat. D4200). Plasmids were then used to transfect HEK Expi293 cells (ThermoFisher, cat. A14527), cultured in a shaking incubator in 8% CO_2_ at 37 °C in BalanCD HEK293 media (Irvine Scientific, cat. 91165-1) supplemented with L-glutamine. Cells were transfected with the plasmids at a 2:1 light chain: heavy chain ratio for IgG-like BiKE. After 5 d, the cell supernatant containing proteins was harvested. IgG-like BiKE was purified with Protein G chromatography. Non-IgG-like constructs were purified with Nickel affinity chromatography. Purity was assessed with FPLC and SDS-PAGE.

### 2.3 Target binding assays

For binding to PD-1+ cells, either EL4, EL4 PD-1^KO^, or primary PD-1^+^ cells were incubated with varying concentrations of PD-1 BiKE for 30 min in PBS at 4°C. Cells were washed, then anti-Rat IgG2a-FITC detection antibody (BioLegend, cat. 405406) was added for 30 min at 4°C. Flow cytometry (BD FACS Canto) was used to detect BiKE-bound cells. For simultaneous binding to PD-1^+^ cells and CD16, PD-1^+^ cells were incubated with varying concentrations of PD-1 BiKE for 30 min in PBS at 4°C, washed, then 150 nM CD16 soluble protein carrying a His tag (AcroBiosystems, cat. CDA-M52H8) was added for 30 min. After washing, anti-His-APC antibody (BioLegend, cat. 36205) was added to cells to detect CD16-bound cells; cells were washed, then flow cytometry was used to determine the quantity of cells bound by PD-1 BiKE and CD16 protein. For simultaneous binding to CD16 ^+^ cells and PD-1, NK cells were incubated with varying concentrations of PD-1 BiKE for 30 min in PBS at 4°C, washed, then 150 nM PD-1 soluble protein carrying a His tag (AcroBiosystems, cat. PD1-M52H3) was added for 30 min. After washing, anti-His-APC antibody (BioLegend, cat. 36205) was added to cells to detect PD-1-bound cells, cells were washed, then flow cytometry was used to determine the quantity of cells bound by PD-1 BiKE and PD-1 protein.

### 2.4 Cell-cell interaction assay

EL4 or EL4 PD-1^KO^ cells were pre-labeled with CFSE (Invitrogen, cat. 65-0850-84); NK cells were prelabeled with eFluor-670 (Invitrogen, cat.65-0840-85). NK cells and either EL4 or EL4 PD-1^KO^ cells were mixed and incubated for 1 h with or without PD-1 BiKE or D-αPD-1 at 37°C. 4% PFA was added after one h to crosslink the cells and fix any cell-cell interactions. Cells were washed, and flow cytometry was used to detect the quantity of CFSE^+^/eFluor-670^+^ double-positive events.

### 2.5 Competitive binding assays

For CD16 competitive binding, NK cells were incubated in tubes with BiKE constructs or D-αPD-1 at 4°C for 30 min. Cells were washed with PBS, then incubated with an anti-CD16-PE antibody (BioLegend, cat. 158004) for 30 min at 4°C. Cells were washed, and flow cytometry was used to quantify the MFIs of each treatment group. For PD-1 binding, PD-1^+^ cells were incubated with BiKE constructs at 4°C for 30 min. Using an anti-PD-1-APC antibody (BioLegend, cat. 135210), the same protocol as above was used to quantify bound proteins. Percent inhibition was calculated with the formula 100% - ((treatment MFI/untreated MFI)*100).

### 2.6 NK cell activation assay

EL4 cells and NK cells were seeded in a 96-well plate at a 2:1 E:T ratio in 200 μL RPMI containing 10% FBS. Concentrations of 0, 3, or 30 nM of PD-1 BiKE were added to respective wells, and plates were incubated 37°C with 5% CO_2_ for 4 h. Cells were transferred to tubes, washed, then resuspended in PBS + 2% FBS. Cells were stained with anti-CD69-BV510 antibody (BioLegend, cat. 104531) and anti-CD3-FITC antibody (BioLegend, cat.100204) for 30 min at 4°C. Cells were washed twice, then analyzed with flow cytometry. Cells that stained negative for CD3 and positive for CD69 were characterized as activated NK cells.

### 2.7 Apoptosis assays

Target cells and primary NK cells were seeded at a 2:1 *E*:*T* ratio in a 96-well plate in 200 μL RPMI containing 10% FBS with varying concentrations of PD-1 BiKE. Cells were incubated at 37°C with 5% CO_2_ for 4 h. Cells were transferred to tubes, washed, and resuspended in Annexin V binding buffer (Biolgened, cat. 422201); anti-CD3-FITC (BioLegend, cat. 100204), Annexin V-APC (BioLegend, cat. 640912), and propidium iodide (Invitrogen, cat. P3566) were then incubated with cells for 30 min at 4°C. Cells were washed twice then analyzed by flow cytometry: cells that stained positive for CD3, and either Annexin V, PI, or both were determined to be apoptotic target cells.

### 2.8 Target cell depletion assays

For EL4/EL4 PD-1^KO^ depletion: target cells and PBMCs were seeded at 20:1 E:T ratios in a 48-well plate in 400 μL RPMI containing 10% FBS and dosed with various concentrations of PD-1 BiKE. Plates were incubated at 37°C in 5% CO_2_. After 24 h, cells were transferred to tubes, washed, and resuspended in PBS containing 2% FBS. Cells were stained with anti-CD3-FITC (BioLegend, cat. 100204) and anti-TCRvβ12-PE (BioLegend, cat. 139703) antibodies. Remaining target cells were quantified using flow cytometry as cells that were TCRvβ12^+^/CD3^+^ double-positive.

For primary T cell depletion: either stimulated or unstimulated primary CD4 T cells were prelabeled with CFSE and seeded with PBMCs at a 20:1 E:T ratio in a 48-well plate μL RPMI containing 10% FBS with or without PD-1 BiKE. Plates were incubated at 37°C in 5% CO_2_. After 24 h, cells were transferred to tubes, washed, and resuspended in PBS containing 2% FBS. Cells were stained with anti-CD4-PE (BioLegend, cat. 100408), washed, then flow cytometry was used to quantify the remaining target cells as those that were CFSE^+^/CD4^+^ double-positive.

### 2.9 *In vivo* efficacy of PD-1 BiKE

C57BL/6 mice were purchased from The Jackson Laboratories. Animal studies were conducted following a protocol approved by the Institutional Animal Care and Use Committee (IACUC) at the University of Utah.

#### 2.9.1 EL4 tumor model

10-week-old female C57BL/6 mice were inoculated with 3x10^6 EL4 cells per mouse via tail vein injection. Two d later, mice were randomly assigned to treatment groups and given either PBS or 100 μg PD-1 BiKE via tail vein injection. 8 d later, the mice were euthanized, and the bone marrow was collected from the femurs of each mouse. Samples were washed, and cells were stained for anti-CD45-PerCP/Cy5.5 (BioLegend, cat. 103132), anti-CD3-FITC (BioLegend, cat. 100204), anti-TCRvβ12-PE (BioLegend, cat. 139703), and anti-PD-1-APC (BioLegend, cat. 135210). Cells positive for all four markers are EL4 cells, which were quantified among total T cells using flow cytometry.

#### 2.9.2 EAE disease model

8-week-old female C57BL/6 mice were subcutaneously injected with 200 μg MOG_35–55_ peptide (ABI Scientific, custom peptide) emulsified in CFA (Sigma Aldrich, cat. F5881). Four and 48 h later, the mice were injected with 0.2 μg pertussis toxin (List Biologicals, #180)) via tail vein injection. Mice were monitored daily for clinical symptoms and scored according to a common standard (43). When a mouse reached a clinical score of 2-3, they were immediately given PBS or 50 μg of PD-1 BiKE via tail vein injection. 48 h after the dose, mice were perfused with saline solution, euthanized, then the brains and spinal cords were collected for analysis. Brains and spinal cords from mice were homogenized, samples were strained through a 70 μm cell strainer, and white blood cells were isolated using a Percoll density gradient. Lymphocytes were washed and stained with anti-CD45-PerCP/Cy5.5 (BioLegend, cat. 103132), anti-CD3-FITC (BioLegend, cat. 100204), anti-PD-1-APC (BioLegend, cat. 135210), anti-CD4-BV510 (BioLegend, cat. 100553), anti-CTLA-4-PE (BioLegend, cat. 106305), anti-CD69-PE/Cy7 (BioLegend, cat. 104511). Flow cytometry was used to analyze the samples from each mouse.

### 2.10 Statistical analysis

Analysis of variance (ANOVA) was used to assess the differences among the means of treatment groups followed by multiple comparisons using the Bonferroni adjustment when dose-response was examined. An additional two-sample comparisons using a one-sided t-test were performed for groups of interest and is reported in the Figures.

## 3 Results

### 3.1 Design, production, and binding verification of mouse-specific PD-1 BiKE

**Figure 1:**
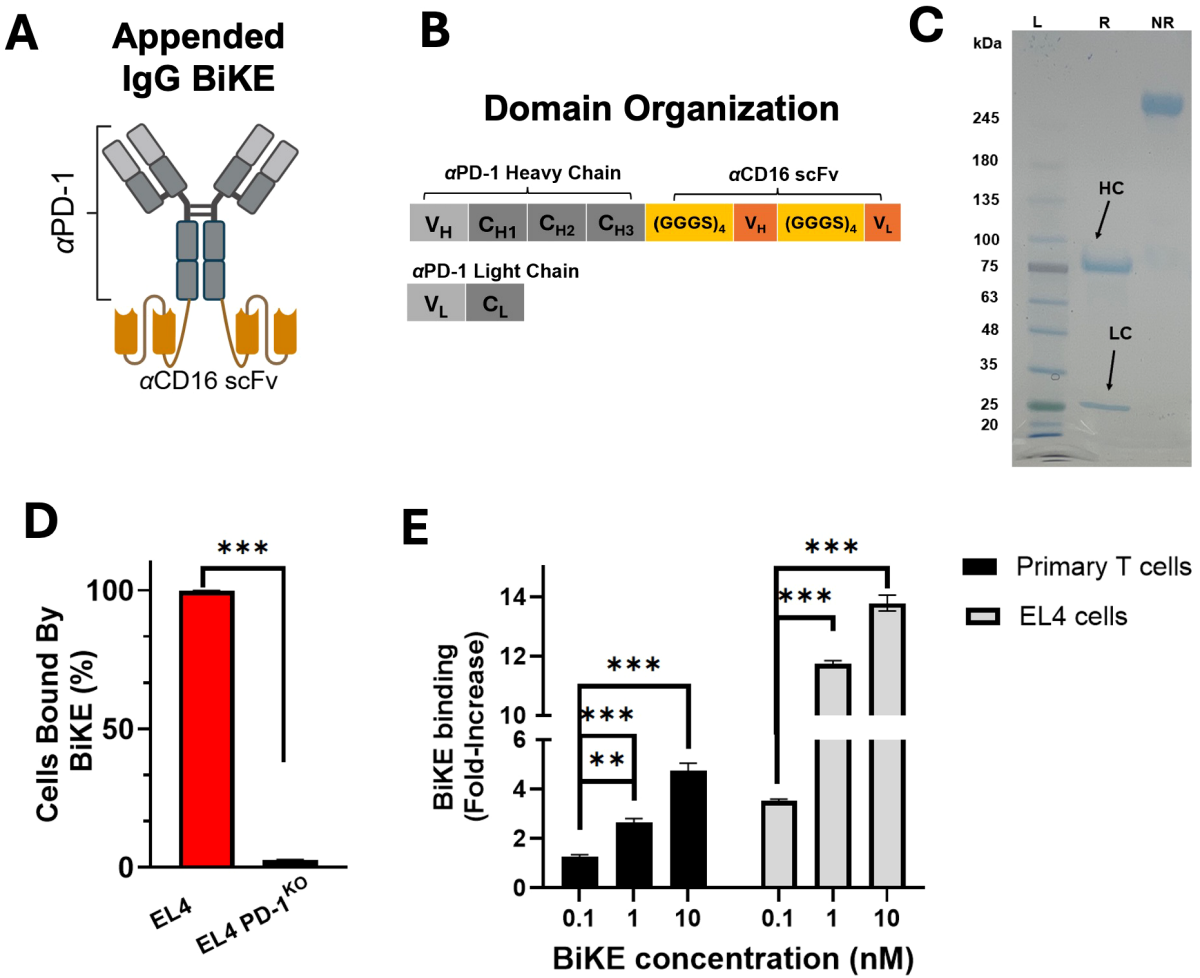
PD-1 BiKE was expressed and binds selectively to PD-1. **(A)** structure of appended IgG BiKE consisting of an αPD-1 backbone and two αCD16 scFvs attached to the Fc via flexible amino acid linkers; **(B)** domain organization of the IgG-like BiKE encoded by the genes of two plasmids used to transfect Expi293 cells; **(C)** SDS PAGE showing BiKE in reducing (R) and non-reducing (NR) loading buffers; in reducing buffer, the heavy chain (HC) shows a band consistent with a protein of 75 kDa, while the light chain (LC) band appears at 25 kDa; the NR and R lanes together demonstrate that the two chains of BiKE are connected via disulfide bonds; **(D)** PD-1 BiKE binds to PD-1^+^ but not PD-1^-^ cells; either EL4 cells or EL4 PD-1^KO^ cells were incubated with BiKE for 30 min, then an anti-Fc antibody was used to detect BiKE-bound cells using flow cytometry; **(E)** BiKE binds to both primary PD-1^+^ cells and EL4 cells in a dose-dependent manner; either primary PD-1^+^ cells or EL4 cells were incubated with varying concentrations of BiKE for 30 min, then an anti-Fc antibody was added and flow cytometry was used to measure the mean fluorescence intensity (MFI) of the cells. Data are mean ± SD, n=5 **p<0.01, and ***p<0.001.

To explore whether the BiKE technology is effective in depleting primary, non-malignant cells in a non-oncology setting, more specifically whether the technology could facilitate the ablation of PD-1^+^ T lymphocytes *in vivo*, we designed a mouse-specific PD-1 BiKE. The PD-1 BiKE was designed in an “appended IgG” format (44) (**Figure 1A**) with bivalent binding capacity to mouse PD-1 and mouse CD16, respectively. The BiKE consists of an αPD-1 backbone attached with two αCD16 scFvs (clone 2.4G2) via flexible peptide linkers. The backbone is composed of the V_L_ and V_H_ of an αPD-1 antibody (clone 1A.12), the constant domains of the rat (kappa) light chain, and the constant heavy domains of rat IgG2a isotype. The BiKE is designed to engage the CD16 receptor of NK cells with high affinity and avidity via the two appended αCD16 scFvs, giving it advantages over a conventional depleting antibody Fc with monovalent, low-affinity engagement. To ensure that CD16 engagement of the BiKE is primarily through the αCD16 scFvs and not through the Fc, rat IgG2a was used as the αPD-1 backbone, since it does not bind strongly to mouse CD16 (45). The design scheme is similar to approved human BiKEs (46–48) that have “muted” Fc regions to avoid inadvertent induction of effector functions through the Fc.

For the production of PD-1 BiKE, two plasmids were designed (**Figure 1B**). The first plasmid encodes the appended heavy chain, which from N to C terminus contains the heavy chain of the αPD-1 backbone, the peptide linker (GGGGS)_3_, the αCD16 V_H_, another linker (GGGGS)_3_, and the αCD16 V_L_ (**Figure 1B**). The second plasmid encodes the light chain of αPD-1. The BiKE has a theoretical molecular weight of approximately 200 kDa and is composed of four polypeptide chains with two distinct lengths: two light chains that are 25 kDa, and two appended heavy chains that are 75 kDa. The size of the four chains and the purity of the BiKE were examined using SDS PAGE (**Figure 1C**). In the sample treated with reducing loading buffer, two bands appeared, one at 75 kDa (appended heavy chain) and one at 25 kDa (αPD-1 light chain); in the sample treated with non-reducing loading buffer, there is only one band with no impurities present. The SDS-PAGE results of the reduced sample confirm that the two types of composite polypeptides of the BiKE are the correct molecular weights, and that the heavy and light chains of the BiKE are connected via disulfide bonds. The final yield of the BiKE was ~6 mg/L of culture.

Following purification, we examined whether the BiKE could selectively bind to PD-1^+^ cells using flow cytometry. When BiKE was incubated with EL4 (PD-1^+^) cells, 99.8% of the cells were bound by BiKE. However, when EL4 PD-1^KO^ cells were incubated with BiKE, fewer than 4% of cells were bound by BiKE (**Figure 1D**), confirming the specificity of the αPD-1 domain of the BiKE.

Next, we examined the binding of several doses of PD-1 BiKE using two different cell types, primary PD-1^+^ T cells and the EL4 cells, which have moderate and high PD-1 expression, respectively (**Supplementary Figure S1**). PD-1 expression on primary T cells was induced with αCD3 and αCD28 stimulation. The primary T cells or EL4 cells were incubated with 0, 0.1, 1, or 10 nM of BiKE and flow cytometry was used to quantify the mean fluorescence intensity (MFIs) of the cells. For the primary cells, at 0.1 nM, there was a 1.2-fold increase in MFI from the condition where there was no BiKE present (**Figure 1E**). At 1nM and 10 nM, the increase was 2.6-fold and 4.7-fold, respectively. Compared to the condition when there was 0 nM BiKE, the MFIs were significantly higher at all three concentrations of BiKE. For the EL4 cells, at 0.1 nM, the MFI increase was 3.5-fold from 0 nM BiKE (**Figure 1E**); at 1 nM an 10 nM, the MFIs increased by 11-fold and 13.7-fold, respectively. Similar to the primary cells, there was a significant increase in BiKE binding to PD-1 at all concentrations compared to when there was no BiKE present. These results highlight that the BiKE binds to PD-1^+^ cells in a dose-dependent manner, and that despite the difference in PD-1 expression, the binding selectivity is consistent between the two cell types.

Altogether, the results show that the produced BiKE assumed a functional configuration that supports its binding to PD-1^+^ cells.

### 3.2 BiKE engages PD-1 and CD16 simultaneously

**Figure 2:**
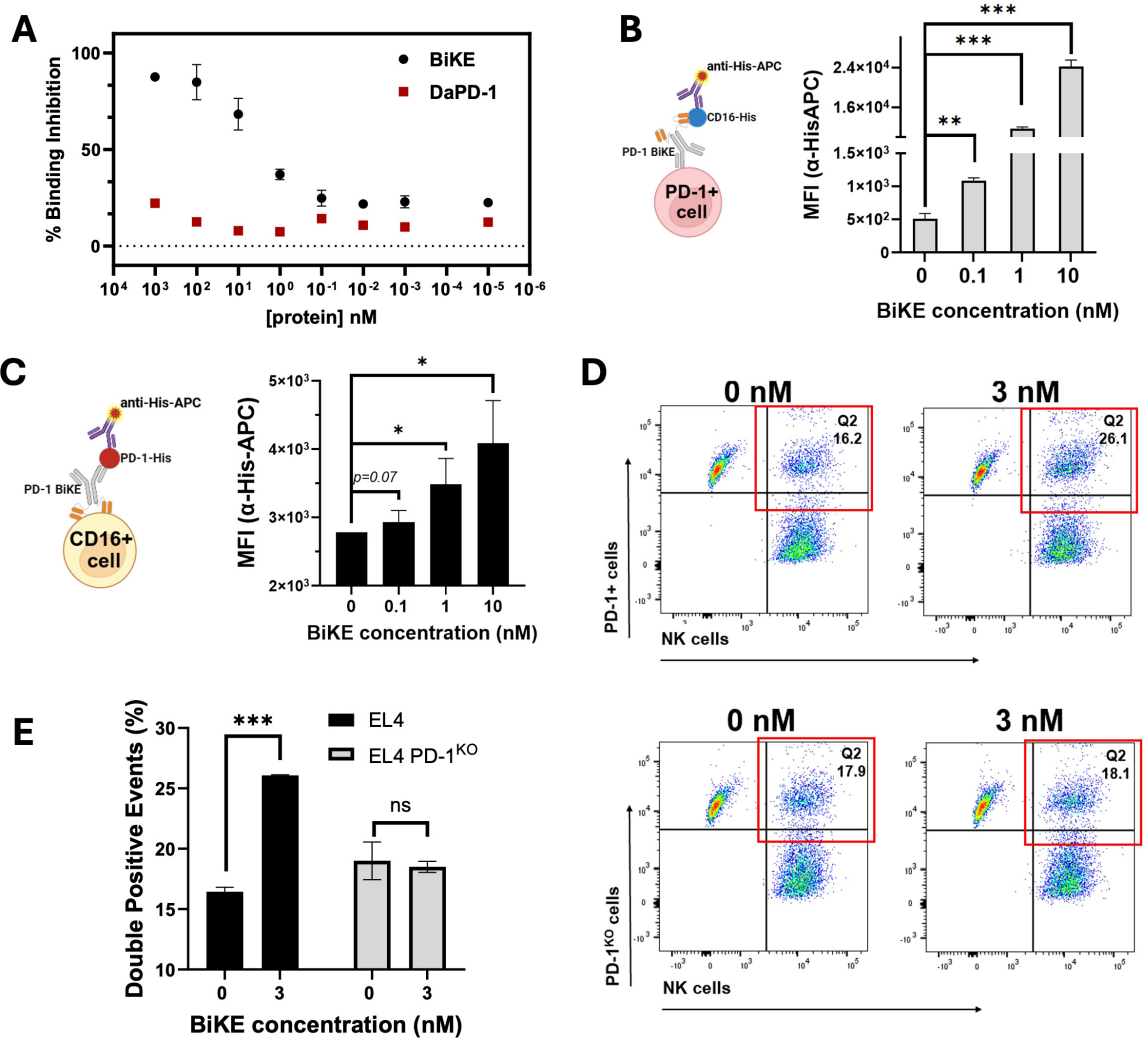
BiKE engages with CD16 and PD-1 simultaneously and bridges PD-1^+^ cells to NK cells. **(A)** competitive binding assay demonstrating the higher affinity of PD-1 BiKE for CD16 compared to a conventional depleting antibody (D-αPD-1); NK cells were incubated with varying concentrations of BiKE or D-αPD-1, washed, then exposed to anti-CD16 detection antibody; MFIs were used to quantify the percentage of binding inhibition; **(B)** BiKE simultaneously binds to PD-1^+^ cells and CD16 and the binding is dose-dependent; EL4 cells were incubated with varying concentrations of BiKE, then exposed to soluble CD16-HisTag, after washing, cells were incubated with an anti-His-APC detection antibody and flow cytometry was used to quantify the MFIs; cells that stained positive for anti-His-APC were considered to be associated with CD16; **(C)** PD-1 BiKE simultaneously binds to CD16^+^ cells and PD-1 in a dose-dependent manner; NK cells were incubated with increasing concentrations of BiKE, then exposed to soluble PD-1-HisTag, after washing cells were incubated with anti-His-APC detection antibody as described in **(B)**; cells that stained positive for anti-His-APC were considered associated with PD-1; **(D)** representative flow cytometry gating demonstrating BiKE binds PD-1^+^ cells and NK cells simultaneously; EL4, or EL4 PD-1^KO^ cells and NK cells were labeled with CFSE or eFluor-670, respectively; the two cells types were mixed and incubated with or without PD-1 BiKE, then PFA was added to fix the cells and secure cell-cell interactions; flow cytometry was then used to quantify the percentage of cell-cell interactions (eFluor-670^+^/CFSE^+^ double-positive events); when incubated with BiKE, there was a greater percentage of double-positive events than when there was no BiKE present; **(E)** bar graph summarizing results from **(D)**; Data are mean ± SD, n=3 *p<0.05, **p<0.01, and ***p<0.001.

Before examining the simultaneous engagement of PD-1 and CD16, we first checked whether the PD-1 BiKE had a higher binding affinity for CD16 compared to our conventional depleting αPD-1 (D-αPD-1). Importantly, PD-1 BiKE was designed with bivalent targeting of CD16 to increase the avidity and affinity interactions with CD16 compared to the monovalent Fc of D-αPD-1. To investigate this, we used a competitive binding assay and found that the PD-1 BiKE exhibited significantly higher binding inhibition at the concentrations tested compared to D-αPD-1 (**Figure 2A**). For example, the PD-1 BiKE achieved 87% binding inhibition at 100 nM, while even the highest concentration (1μM) of D-αPD-1 only achieved 22.2% inhibition. From the resulting nonlinear regressions, the PD-1 BiKE was determined to have a dissociation constant of 3.87 nM, while the D-αPD-1 dissociation constant was >1 μM. This result supports that the PD-1 BiKE has a higher binding affinity for CD16 compared to a conventional depleting antibody.

Apart from higher binding affinity for CD16, a critical prerequisite of the PD-1 BiKE function is that it can engage PD-1^+^ T cells and CD16^+^ NK cells simultaneously. To investigate this, we first used a cell-based ELISA assay involving PD-1^+^ cells, the PD-1 BiKE, and a protein pair that can detect BiKE on the cell surface (**Figure 2B**). Using PD-1^+^ T cells, we first examined whether PD-1 BiKE that is bound to PD-1^+^ cells can also bind to CD16 (**Figure 2B**). At 0.1 nM, the average MFI of the cells was 1088, a two-fold increase from the baseline MFI (**Figure 2B**). At 1 nM and 10 nM, the MFIs increased to 11691 and 24228, a 22- and 47-fold increase, respectively. Compared to the condition where there was no BiKE present, all three concentrations resulted in greater association of CD16 to the PD-1^+^ T cells, indicating that PD-1 BiKE can bind to PD-1^+^ T cells and CD16 at the same time, and this binding is concentration-dependent.

Next, we used a similar assay to determine whether BiKE bound to NK cells could simultaneously engage PD-1 (**Figure 2C**). At 0.1 nM PD-1 BiKE, the cells had an average MFI of 2900, a 1-fold increase from baseline (**Figure 2B**); at 1 nM the MFI increased to 3500, a 1.25-fold increase from baseline, and at 10 nM the MFI increased to 4089, a 1.45-fold increase from baseline. These results indicate that when BiKE is bound to NK cells, it can also bind to PD-1, while the fold-changes demonstrate that the binding is concentration-dependent. Together, the cell-based assays support that with increasing BiKE, there are increasing opportunities for engagement of both cellular targets.

The next important feature of the BiKE is that it can bind both PD-1^+^ T cells and NK cells at the same time to facilitate the interaction of the two cell types. Pre-labeled target T cells and NK cells (eFluor-670 and CFSE, respectively) were incubated together with or without PD-1 BiKE, then PFA was added to fix the cells and solidify any cell-cell interactions that had formed. Flow cytometry was then used to quantify the cells that are positive for both eFluor-670 and CFSE (double-positive events) (**Figure 2D**). When PD-1 BiKE was added, the fraction of double-positive events was 26.07%, on average, compared to 16.01% when there was no BiKE present (**Figures 2D, E**). Importantly, the addition of PD-1 BiKE did not change the fraction of double-positive events when PD-1^KO^ cells were used as the target cells (**Figures 2D, E**), indicating that the induced interaction is PD-1-dependent. In line with our hypothesis of BiKE promoting greater NK cell-PD-1^+^ cell interaction compared to a conventional depleting antibody, the addition of 30 nM PD-1 BiKE resulted in a 38.8% increase in double-positive events compared to 30 nM D-αPD-1 (**Supplementary Figures S2A, B**).

Altogether, the results indicate that PD-1 BiKE can bind to both PD-1 and CD16, and promotes the interaction of the two cell types.

### 3.3 PD-1 BiKE activates NK cells and mediates depletion of PD-1^+^ T cells

**Figure 3:**
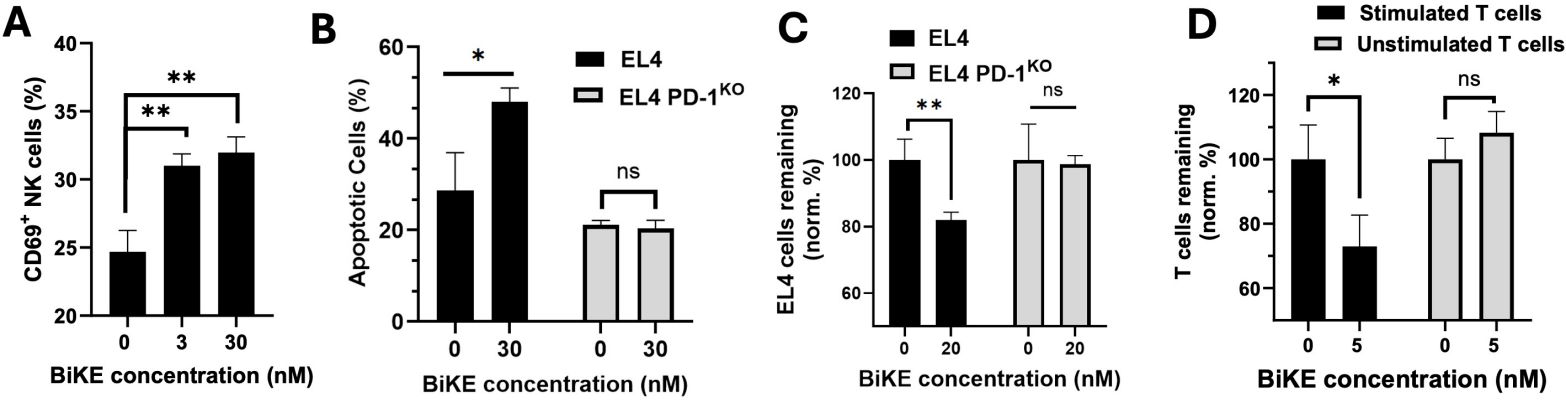
BiKE induces NK cell activation and depletion of PD-1^+^ T cells. **(A)** BiKE induces activation (CD69 expression) of NK cells when co-cultured with PD-1^+^ T cells; NK cells were incubated with EL4 cells for 4 hours with or without BiKE and CD69 expression on NK was quantified using flow cytometry; when incubated with BiKE, there was a greater percentage of CD69^+^ NK cells compared to when there was no BiKE present; **(B)** PD-1 BiKE induces apoptosis in PD-1^+^ but not PD-1^-^ T cells when co-cultured with NK cells; either EL4 or EL4 PD-1^KO^ cells were seeded with NK cells for 4 h with or without BiKE, then, the fraction of apoptotic EL4 or EL4 PD-1^KO^ cells was quantified by staining with annexin V and PI; **(C)** BIKE induces the depletion of PD-1^+^ but not PD-1^-^ T cells; either EL4 or EL4 PD-1^KO^ cells were co-cultured with PBMCs for 24 h in the presence or absence of PD-1 BiKE and flow cytometry was used to detect the remaining number of target cells; when EL4 cells were incubated with BiKE the fraction of remaining T cells was reduced compared to when there was no BiKE present; when PD-1^KO^ cells were incubated with BiKE, there was no reduction in T cells; **(D)** PD-1 BiKE induces the depletion of primary PD-1^+^ T cells; stimulated primary T cells or unstimulated primary T cells were prelabeled with CFSE, then co-cultured with PBMCs for 24 hours with or without BiKE; flow cytometry was then used to quantify the remaining CFSE+ target cells; Data are mean ± SD, n=3 *p<0.05, and **p<0.01. Percentages shown in **(C, D)** are normalized to respective 0 nM BiKE groups.

To investigate whether the BiKE could induce NK cell activation, and whether the extent of activation is concentration-dependent, we co-cultured PD-1^+^ T cells with NK cells with 0 nM, 3 nM, or 30 nM PD-1 BiKE, then quantified the numbers of activated NK cells via CD69 expression. When 3 nM BiKE was used, 31% of NK cells were CD69^+^ (**Figure 3A**); when 30 nM BiKE was used, 32.9% of NK cells were CD69^+^. Both concentrations of BiKE resulted in a significant increase from an average of 23.7% CD69^+^ NK cells when there was no BiKE present (**Figure 3A**), though the concentration effect was not dramatic between the two concentrations tested.

Next, we wanted to examine whether the BiKE could increase NK cell-mediated apoptosis in PD-1^+^ T cells. Here, we co-cultured either EL4 cells or PD-1^KO^ cells and NK cells with or without PD-1 BiKE, then used flow cytometry to quantify the fraction of apoptotic target cells. When the EL4 cells were dosed with PD-1 BiKE, the fraction of apoptotic cells increased to 48.05% from 28.65% when there was no BiKE present (**Figure 3B**). However, the BiKE did not increase apoptosis of PD-1^KO^ cells in the co-culture with NK cells. These results indicate that PD-1 BiKE can increase the NK-mediated apoptosis of PD-1^+^ T cells, and that this effect is PD-1-dependent.

Further, we examined whether the BiKE could induce the depletion of PD-1^+^ T cells. Either EL4 cells or PD-1^KO^ cells were co-cultured with PBMCs with or without PD-1 BiKE, then flow cytometry was used to detect the number of target cells remaining in the co-culture after 24 hours. When EL4 cells were given PD-1 BiKE, the fraction of EL4 cells remaining was 81%, a 19% decrease compared to that of the group where 0 nM BiKE was used (**Figure 3C**). However, when the PD-1^KO^ cells were cultured with NK cells and PD-1 BiKE, there was no salient reduction in target cells (**Figure 3C**).

Next, we expanded the investigation of this question to primary T cells, which have lower and heterogeneous PD-1 expression. First, primary CD4 T cells were isolated from mouse splenocytes, then cultured in stimulating conditions with αCD3 and αCD28 antibodies to induce PD-1 expression. T cells were also left unstimulated as a negative control (**Supplementary Figure S3**). When the stimulated T cells were dosed with PD-1 BiKE, the fraction of T cells remaining after 24 hours was 72.99%, indicating a 27.1% reduction in PD-1^+^ cells from the group where there was no BiKE present (**Figure 3D**). On the other hand, the presence of PD-1 BiKE did not decrease the number of unstimulated cells remaining (**Figure 3D**).

These results show that the PD-1 BiKE can activate NK cells and selectively promote NK cell-mediated apoptosis and depletion of PD-1^+^ cells. Importantly, this depleting effect was not only observed on PD-1^+^ cell line, but on primary PD-1^+^ T cells that are relevant to autoimmunity.

### 3.4 PD-1 BiKE depletes PD-1^+^ T cells *in vivo*

**Figure 4:**
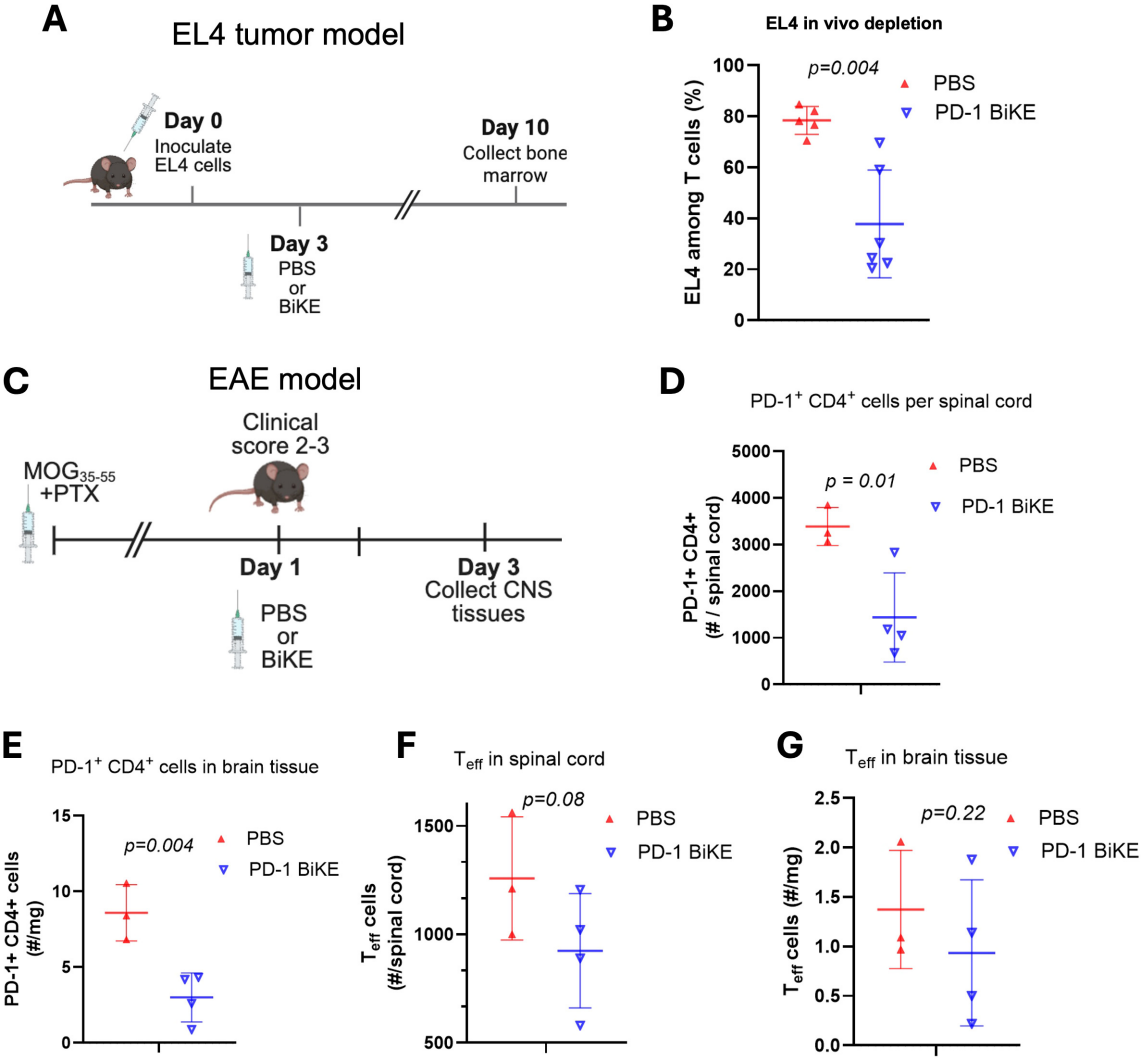
PD-1 BiKE depletes PD-1^+^ cells *in vivo*. **(A)** experimental timeline of EL4 tumor model; B6 mice were inoculated with 3x10^6 EL4 cells on day 0, on day 3 mice were either treated with PBS or BiKE, on day 10 or 11, mice were euthanized and bone marrow was collected for analysis via flow cytometry; **(B)** PD-1 BiKE reduces the fraction of EL4 cells in the bone marrow of inoculated mice compared to those treated with PBS; collected bone marrow was processed and stained with antibodies against CD45, CD3, TCRvβ12, and PD-1; fractions shown are TCRvβ12^+^ PD-1^+^ double positive cells among total CD3^+^ cells; **(C)** experimental timeline of the EAE disease model; EAE was induced in B6 mice; when a mouse reached a clinical score of 2-3, they immediately received one IV injection of either PBS or PD-1 BiKE; two days after the injection, mice were euthanized and the brain and spinal cords were collected for analysis via flow cytometry; **(D)** mice treated with BiKE had lower numbers of PD-1^+^ CD4^+^ cells per spinal cord, on average, compared to PBS-treated mice; **(E)** mice treated with BiKE had lower numbers of PD-1^+^ CD4^+^ cells per mg of brain tissue compared to mice treated with PBS; **(F)** CD69^+^/CTLA-4^+^ double positive cells were used to identify effector T cells (Teff) in the spinal cord; on average, there were fewer Teff cells in the spinal cords of mice treated with PD-1 BiKE compared to PBS; **(G)** the average number of Teff per mg of brain tissue was reduced in mice treated with PD-1 BiKE compared to PBS. Data are mean ± SD, n=5/6 **(B)**; n= 3/4 **(D-G)**.

To explore the use of PD-1 BiKE *in vivo*, we first used EL4 cells as target PD-1^+^ T cells. C7BL/6 mice were inoculated with EL4 cells and given either PBS or 100 ug PD-1 BiKE based on our previous study targeting PD-1^+^ cells (49) and reported doses of IgG-like bispecific antibodies (50, 51); on day 10, the bone marrow was collected for analysis because EL4 cells, if not depleted, become detectable in the bone marrow at this time point (49) (**Figure 4A**). In mice treated with PBS, the average fraction of EL4 cells in the bone marrow was 78.9%. In mice treated with PD-1 BiKE, this fraction dropped by approximately half, to 37%, on average (**Figure 4B**). This result indicated that the killing effect of PD-1 BiKE was sustained and that the BiKE could induce the depletion of PD-1^+^ cells *in vivo*, a critical step towards the depletion of primary effector lymphocytes found in autoimmune diseases.

The next important question was whether the PD-1 BiKE could deplete primary PD-1^+^ T cells. We used the experimental autoimmune encephalomyelitis (EAE) disease model since PD-1^+^ CD4 T cells are found in high numbers in the brains and spinal cords of EAE mice (34). We induced EAE using MOG_35–55_ peptide, then waited until the mice reached a clinical score of 2–3 based on data showing that is when the highest number of PD-1^+^ T cells are found in the brain and spinal cord (**Supplementary Figure S4**). The mice were given one dose of either PBS or PD-1 BiKE (49–51) (**Figure 4C**). Two days later, the brains and spinal cords were collected for flow cytometry analysis.

First, we examined the effect of PD-1 BiKE using the number of PD-1^+^ CD4 T cells as a metric. In mice treated with PBS, the average number of PD-1^+^ CD4 T cells in the spinal cord was 3,389. In mice treated with PD-1 BiKE, that average was reduced by approximately 57%, to 1,438 PD-1^+^ CD4 T cells per spinal cord (**Figure 4D**), indicating the depletion of PD-1^+^ cells in the spinal cord via PD-1 BiKE. Next, we quantified the number of PD-1^+^ CD4 cells in the brains. Here, we found that the PBS-treated mice had 8.5 PD-1^+^ CD4 T cells per mg of brain tissue, on average. In the BiKE-treated mice, this average number dropped by 65% to 3.0 (**Figure 4F**), further indicating the depletion of PD-1^+^ cells via PD-1 BiKE. The goal of depleting PD-1^+^ cells is to reduce the number of effector T (Teff) cells in the spinal cord, for which we used CD69^+^/CTLA-4^+^ double-positive expression as a marker. Here, we found that the average number of Teff cells was also reduced in both the spinal cords and brains of the BiKE-treated mice, compared to the PBS-treated mice (**Figures 4F, G**). The BiKE-treated mice had, on average, 924.3 Teff in the spinal cord compared to 1,257.7 for the PBS-treated mice, while the BiKE-treated mice had 0.935 Teff/mg in the brain compared to 1.4 Teff/mg for PBS-treated mice, though the reductions did not reach statistical significance.

Altogether, the results demonstrate that PD-1 BiKE can be used to deplete PD-1^+^ cells *in vivo*. Importantly, PD-1 BiKE can deplete primary PD-1^+^ T cells in an autoimmune disease model, supporting the use of the BiKE technology in a new disease context.

### 3.5 Non-IgG-like PD-1 BiKEs bind to both cellular targets

Since the IgG-like BiKE was found effective to deplete PD-1^+^ cells, we expanded our exploration using the BiKE technology in a non-oncological setting by generating two non-IgG-like BiKEs. The efforts were warranted as it is unknown whether IgG-like or non-IgG-like BiKEs are advantageous in the context of depleting non-malignant cells, while the answer may be entirely case-dependent.

**Figure 5:**
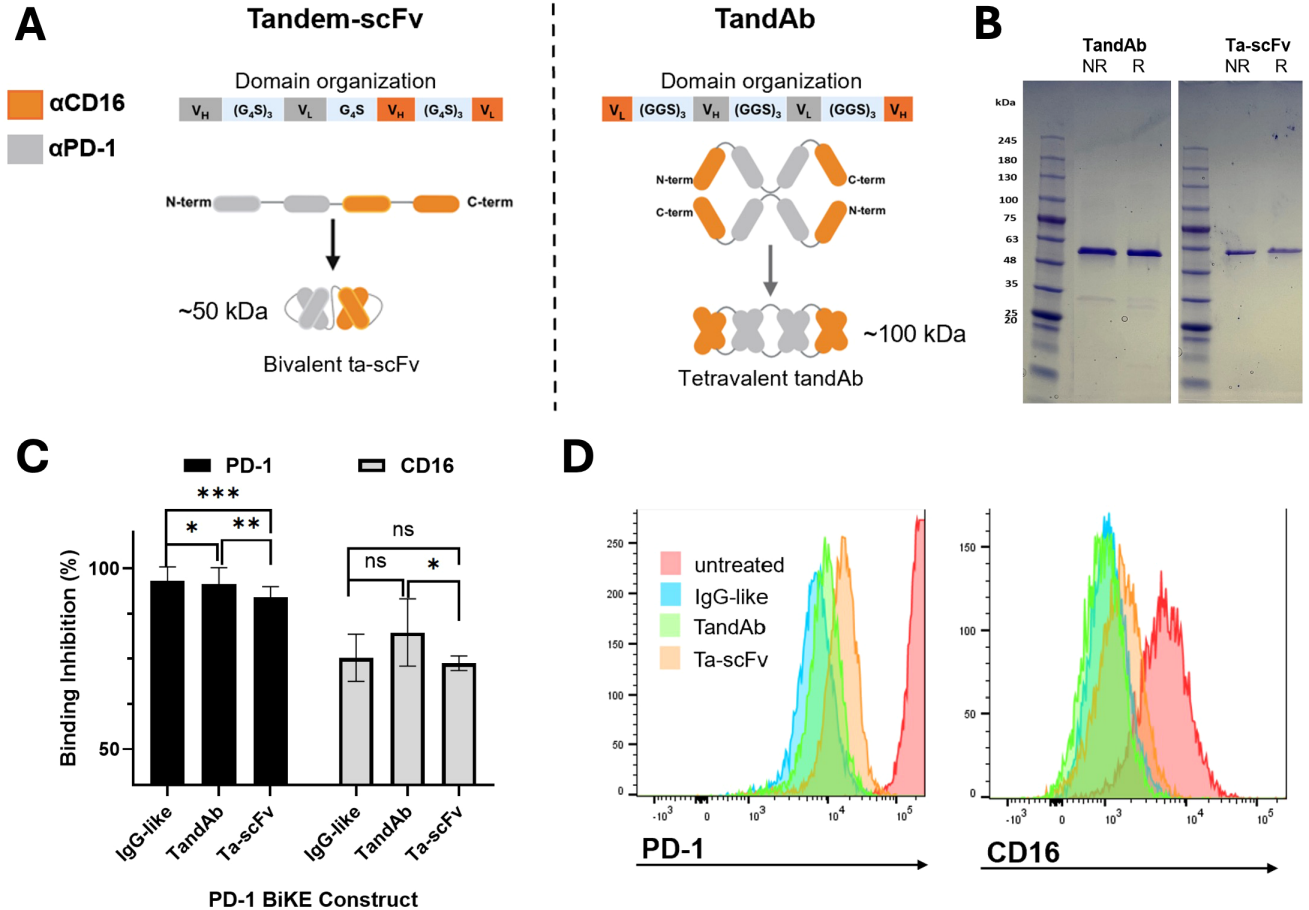
Non-IgG-like PD-1 BiKE constructs bind to PD-1^+^ cells and NK cells. **(A)** Illustrations of two BiKE constructs in the tandem-scFv (ta-scFv) and TandAb formats; Left: domain organization of the ta-scFv construct encoded by the gene of one plasmid used to transfect Expi293 cells, graphic showing the linear polypeptide structure from N to C terminus, and assembled bivalent protein structure; Right: domain organization of the TandAb construct encoded by the gene of one plasmid used to transfect Expi293 cells; graphic showing linear polypeptide structure from N to C terminus that self-assembles in a head-to-tail configuration to make the final, tetravalent BiKE structure; anti-CD16 domains represented by orange color and anti-PD-1 domains represented by grey color for both constructs; **(B)** SDS PAGE of purified TandAb (left) and ta-scFv (right) constructs in either reducing (R) or non-reducing (NR) loading buffers; the two polypeptide chains of the TandAb construct separate and migrate to 50 kDa in both R and NR loading buffers; the Ta-scFv construct migrates to 50 kDa in both R and NR conditions; **(C)** competitive binding assay demonstrates three PD-1 BiKE constructs binding to PD-1 and CD16; for PD-1, the IgG-like BiKE has the greatest percentage of binding inhibition, followed by the TandAb and ta-scFv constructs respectively; for CD16, the TandAb has the greatest percentage of binding inhibition, followed by IgG BiKE and ta-scFv, respectively; **(D)** representative flow cytometry histograms showing binding inhibition to PD-1^+^ (EL4) cells (left) and CD16^+^ (NK) cells (right) by three PD-1 BiKE constructs. Data are mean ± SD, n=3 *p<0.05, **p<0.01, and ***p<0.001, ns, not significant.

One BiKE is in the tandem-scFv (ta-scFv) format (7) (**Figure 5A**). The BiKE is designed to be bivalent, with one binding domain for PD-1 and one binding domain for CD16 (**Figure 5A**). This BiKE is composed of a single polypeptide that contains the V_H_ and V_L_ of αCD16, and the V_H_ and V_L_ of αPD-1, connected by flexible peptide linkers. The second BiKE is in the tandem diabody (TandAb) format (7). The BiKE is designed to be tetravalent, with two binding moieties for PD-1 and two for CD16. The BiKE is composed of two dimerized polypeptide chains that each contain the V_L_ of αCD16, V_H_ and V_L_ of αPD-1, and the V_H_ of αCD16, connected by short amino acid linkers (**Figure 5A**). The polypeptide chains dimerize in a head-to-tail configuration to assemble the final tetravalent structure. While both BiKE structures are designed to engage PD-1 and CD16 simultaneously, the TandAb may exhibit increased binding due to its bivalent binding capacity for both cellular targets.

Both BiKEs were expressed in HEK293 cells. For the ta-scFv BiKE, the cells were transfected with one plasmid encoding the gene for the polypeptide (**Figure 5A**). The ta-scFv has a theoretical molecular weight of 50 kDa. For the TandAb BiKE, the cells were transfected with one plasmid encoding the gene for the single polypeptide. After two polypeptides dimerize, the theoretical molecular weight of the protein is 100 kDa (**Figure 5A**). Both PD-1 BiKEs contain a His tag on the C terminus and were purified using nickel affinity chromatography. SDS PAGE using both reducing and non-reducing loading buffers was used to assess the purity and molecular weights of the polypeptide chains of both PD-1 BiKEs. In both reducing and non-reducing buffers, the TandAb BiKE produces one band consistent with a molecular weight of 50 kDa, (**Figure 5B**), demonstrating that there are no disulfide bonds between the two polypeptide chains. The presence of only one band indicates that the BiKE is pure. Similarly, for the ta-scFv BiKE, both reducing and non-reducing buffers only produce one band consistent with a 50 kDa protein, as it is a single peptide chain, also with no significant impurities present. (**Figure 5B**). Together, the reducing and non-reducing conditions indicate that the polypeptide chains of both BiKEs are the correct molecular weights and that the products are pure.

We investigated whether the two non-IgG-like BiKEs could bind to PD-1 and CD16 and compared them to the IgG-like BiKE using a competitive binding assay (**Figures 5C, D**). As expected, the TandAb BiKE demonstrated significantly stronger binding inhibition to both PD-1 and CD16 compared to the ta-scFv BiKE, due to its tetravalent structure with two binding domains for each target. For PD-1, the TandAb achieved 95.6% binding inhibition, while the ta-scFv achieved 92.0% inhibition (*p<0.01*). For CD16, the TandAb achieved 82.3% inhibition, and the ta-scFv only achieved 73.7% (*p<0.05)*. The TandAb achieved comparable levels of binding inhibition to the IgG-like BiKE for PD-1 (95.6% vs 96.5%, respectively) and for CD16 (82.3% vs. 75.2%, respectively). However, the ta-scFv demonstrated lower levels of inhibition for both targets, 92.0% for PD-1 and 73.7% for CD16, as compared to the tetravalent BiKE configurations, whether it was IgG-like or non-IgG-like. These results indicate that the design of the three BiKE constructs is directly reflected by their capacity to bind each target.

Altogether, the results show that the purified non-IgG-like PD-1 BiKE constructs are the correct molecular weights and bind to both cellular targets.

## 4 Discussion

To make translatable biologics for PD-1^+^ cell depletion, we leveraged the BiKE technology, which to date has been used extensively for anti-cancer therapy, but not for autoimmune disease treatment. In this study, we expanded the BiKE investigation by generating a mouse-specific PD-1 BiKE that was evaluated for its capacity to deplete PD-1^+^ T cells *in vivo* in a non-oncology setting.

While conventional depleting antibodies have long been used for the depletion of both cancer cells and primary cells (e.g., αCD20 for B cell cancers and multiple sclerosis) (52, 53), BiKEs offer several advantages that have made them a promising approach to improve these therapeutics. For example, Fc region of conventional antibodies binds to CD16 with low affinity and low selectivity, requiring a high number of antibodies to bind to the target cell surface before inducing antibody-dependent cellular cytotoxicity (ADCC) (54). However, the emerging BiKE technology has provided a way to target CD16 with high affinity and specificity to induce ADCC more efficiently. Further, with many formats of BiKEs, more binding domains can be incorporated into one molecule compared to a conventional Fc, providing more CD16 binding opportunities per molecule (i.e., tetravalent BiKEs) (5, 55). Indeed, this study demonstrated that the tetravalent IgG-like BiKE targeting CD16 had a higher binding affinity for CD16 on NK cells compared to our conventional D-aPD-1 and promoted more NK cell-PD-1^+^ cell-cell interactions (**Figure 2**), supporting this BiKE advantage. On the other hand, an advantage of conventional antibodies is their simple and reliable production process, which is easily scaled for clinical use. While most BiKEs can also be expressed in mammalian cells, the non-native structures encompassing atypically long or unstable polypeptides can make both production and purification more challenging (56). Another advantage of conventional depleting antibodies is their extended half-lives. As the BiKE technology is novel, there is less information regarding the specific pharmacokinetics of each format, though in the case of IgG-like BiKEs, reported half-lives have been similar to conventional antibodies (57). With the production of D-αPD-1, an IgG-like and two non-IgG-like PD-1 BiKEs, this study provides four representative constructs that can be used to investigate important questions regarding differences between BiKEs and conventional antibodies. Through comparisons in the production, efficacy, and pharmacokinetic parameters of the constructs, we may elucidate which qualities are most beneficial for the depletion of primary cells in autoimmunity.

A major difference when engineering PD-1 BiKEs for effector T cell depletion versus cancer cell depletion is the target antigen expression. Effector cells typically express lower quantities of PD-1 compared to cancer cells. For instance, the EL4 cell line, derived from T cell lymphoma, expresses ~75,000 PD-1 molecules per cell, whereas primary mouse T cells express only 2,500 per cell (unpublished data). Additionally, the PD-1 expression on effector cells is dynamic and can appear at different levels depending on the stage of activation (e.g., PD-1 expression is upregulated on exhausted T cells) (32, 58, 59), making the depleting target somewhat challenging to capture. Because of this dynamic expression, the killing mechanism of a biologic must be efficient. Although our previous immunotoxins offer a simple working mechanism for PD-1^+^ cell depletion (37, 38), they present challenges for clinical translation. Thus, we leveraged the BiKE technology to create a clinically appealing therapy that is also efficacious. Previous BiKEs have shown success in depleting cancer cells with overexpression (e.g., HER2 over-expressing cancers) (11, 60), but it was unknown whether the technology could be used to deplete the primary cells, especially those with limited antigen expression. Though there is a reported CD16-targeting BiKE that depletes HIV-infected CD4 cells with transient gp41 expression (6), the study was limited to *in vitro* validation. Here, we showed that the PD-1 BiKE could deplete high-expressing PD-1^+^ T cells both *in vitro* and *in vivo*, and importantly, that it could deplete lower-PD-1 expressing primary T cells in the EAE disease model. Therefore, this study demonstrates that BiKEs can be used to deplete both cancer cells and primary cells, even given the inherent differences in antigen expression between the two cell types.

In terms of depleting primary T cells versus cancer cells, there are advantages and disadvantages for BiKEs targeting each cell type. While the BiKE mechanism of activating NK cells is the same for both cancer cells and primary cells, there are differences in the two cell types that present unknowns about the translation of the technology. NK cells are innate responders that have several mechanisms for detecting malignant cells, potentially enhancing their susceptibility to BiKE-mediated killing (61). For example, malignant cells have downregulated MHC I expression, which is recognized by NK cells and leads to their activation against the cells. However, primary cells express high levels of MHC I, which are recognized by inhibitory receptors on NK cells and suppress their activation (62). Similarly, tumorigenic cells overexpress death receptors such as Fas and death receptors 4 and 5 (DR4/5), which bind ligands on NK cells and induce their activation, while primary cells have minimal expression of such receptors to protect from NK attacks (19). Conversely, there are advantages for using BiKEs to deplete primary cells that may make them superior BiKE targets. For example, it has been demonstrated that cancer cells can develop resistance to pore formation via perforin and pro-apoptotic calcium influx that is caused by NK cell activation through CD16 (19). With this consideration, it is likely that cancer cells could be more resistant to BiKE-mediated cytotoxicity compared to primary cells. Furthermore, when applying BiKEs to cancer or primary cells, the quantity of target cells that need to be depleted is an important factor. A hallmark of cancer cells is their uncontrolled proliferation, making them very challenging to completely ablate. For this reason, primary cells may be easier to deplete using BiKEs, simply due to their slower and less prolific expansion (63). In fact, the *in vitro* work presented in this study (**Figure 3**) shows that the PD-1 BiKE reduced the primary PD-1+ T cells by a greater percentage than the EL4 cell line, possibly due to the rapid proliferation of cancer cells in the tested 24-hour time frame. Our results from this study demonstrate that there is tremendous potential, and possible advantages, for the translation of this technology from cancer cell depletion to primary cell depletion.

Lastly, the number, proximity, and availability of NK cells in the two different immune environments may be very different. NK cells are a good candidate for BiKEs because the majority (~90%) are mature, CD16-expressing cells that are readily activated with no prior sensitization (64). This is useful for both autoimmunity and cancer treatment. Though NK cells are early responders that get mobilized to tumor environments, it is common that in later stages of solid tumor growth, NK cell infiltration is minimal, making the availability of NK cells for BiKE-mediated activation challenging in these cases (62). For this reason, hematological cancers have been a larger focus of BiKE development, since certain cancers will likely have the chance to encounter BiKE and NK cells in the secondary lymphoid organs or the blood (65, 66). This is an advantage of translating the BiKE technology to the context of autoimmune disease, especially for the depletion of PD-1^+^ cells. Indeed, we showed in this study that the PD-1 BiKE could deplete the T cell lymphoma that grows in the bone marrow and primary T cells *in vivo.* Though the presence of NK cells in autoimmune disease tissue may vary by disease, we found that NK cells were present in both the brains and spinal cords of EAE mice (**Supplementary Figure S5**) similar to other reports (67, 68), which may allow for BiKE-mediated T cell depletion at the site of inflammation in the CNS of these mice.

Our study demonstrates the capacity of the BiKE technology in the new disease context of autoimmunity. While some bispecific T cell engagers have been explored for their use in autoimmune disease (e.g., blinatumomab for CD19 depletion in MS) (24), these therapies still confer large lymphocyte subset depletion that we are trying to avoid. Similar to current therapies such as ocrelizumab and teplizumab, these T cell engagers indiscriminately deplete lymphocytes, whether they are autoreactive or not. Such therapies are the cause of long-term lymphopenia in patients that elevates their susceptibility to infections and certain malignancies (28). On the other hand, PD-1 is only expressed by activated effector lymphocytes and not naïve cells (32). Hence, the specific depletion of PD-1^+^ cells by the BiKE can reduce the number of activated lymphocytes and slow autoimmune disease progression, but preserve healthy, naïve lymphocyte repertoires. Importantly, these cells can later become activated lymphocytes upon immune stimulation by infections or malignant cells once the BiKE is cleared. Even though the depletion of PD-1^+^ cells may cause acute immunosuppression while BiKE(s) are in circulation, this suppression will end once the BiKE is cleared. Further, the BiKE targeting PD-1 has the opportunity to deplete both T and B lymphocytes that participate in autoimmune attacks, as opposed to just one cell type, giving it distinct advantages over existing monoclonal and bispecific therapies.

A BiKE targeting PD-1 as a tool for effector cell depletion in autoimmunity is a novel step with many potential avenues to explore. Because the translation of the BiKE technology to autoimmunity is new, there are many unknowns regarding what format may be most effective, given the potential differences in half-life and tissue penetration (69). With this and the success of the IgG-like BiKE, we further developed two non-IgG-like PD-1 BiKEs, assuming tandem-scFv and tandem diabody formats. Each BiKE demonstrated binding to both PD-1 and CD16, and the binding was reflective of their valencies for each cellular target (**Figure 5**). Thus, the non-IgG-like BiKEs will be used to further investigate the depletion of PD-1^+^ cells in future studies.

Altogether, our results from PD-1 BiKE demonstrate the expansion of the technology into a broader range of diseases beyond oncological settings. While the current study supports the use of the BiKE technology as a tool for primary cell depletion, it opens the door for more investigations, invaluable to the development of safer and more precise autoimmune disease therapies.

## Data Availability

The original contributions presented in the study are included in the article/**Supplementary Material**, further inquiries can be directed to the corresponding authors.
