# Supplementary material for: PD-1 Bispecific Killer Engager (PD-1 BiKE) effectively depletes effector T lymphocytes in experimental autoimmune encephalomyelitis
Source: Front Immunol. 2025 Aug 13;16:1644903. doi: 10.3389/fimmu.2025.1644903 (PMC12380559; doi:10.3389/fimmu.2025.1644903)
Supplement: Supplementary file 1 [file Image1.pdf]

Supplementary Material

1.1 Supplementary Figures

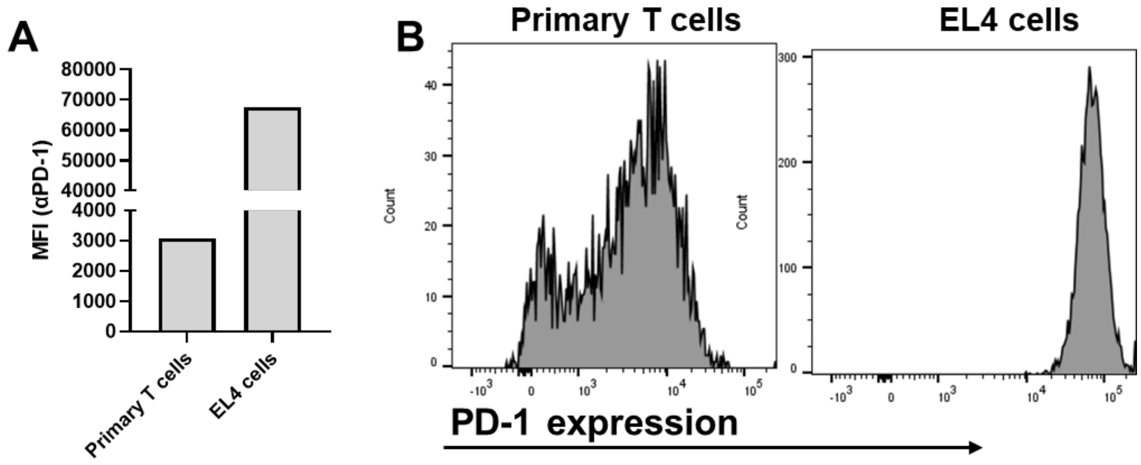

Figure S1. Differences in PD-1 expression between primary T cells and the EL4 cell line. A) Graph showing the MFIs of primary T cells stimulated with αCD3 and αCD28 antibodies vs. the EL4 cell line; B) Flow cytometry histograms showing the PD-1 expression of the two cell types; the stimulated primary T cells have heterogenous and lower PD-1 expression compared to EL4 cells which have higher, uniform PD-1 expression.

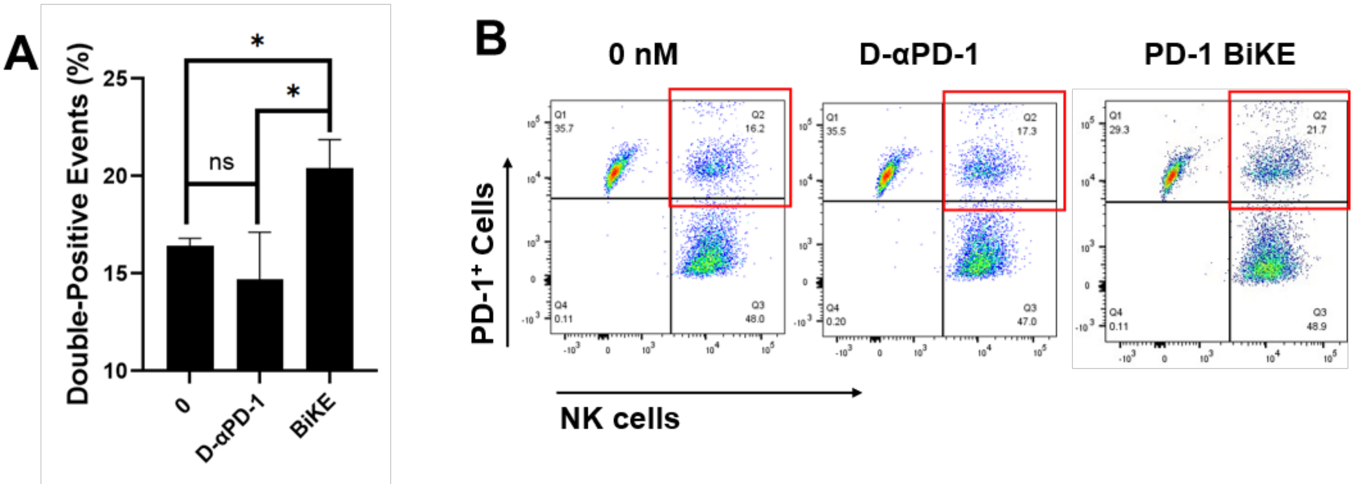

Figure S2. PD-1 BiKE induces greater cell-cell interactions than D-αPD-1. A) Percentages of double-positive events when incubated with 0 nM protein, 30 nM D-αPD-1, or 30 nM PD-1 BiKE; B) Flow cytometry gating showing the percentages of double-positive events when incubated with different proteins. **p<0.05, ns=not significant.*

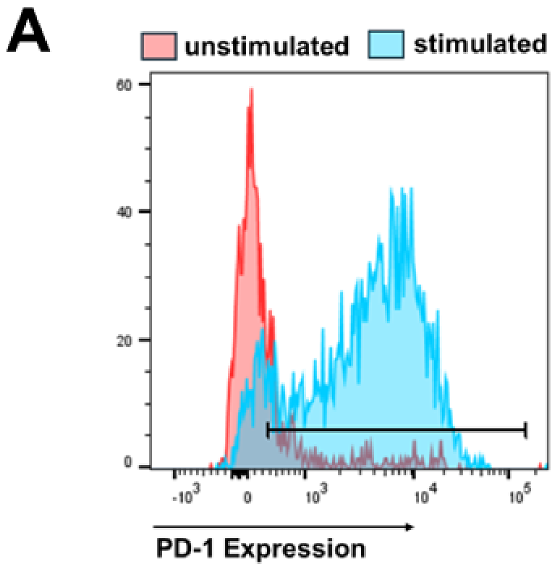

Figure S3. PD-1 expression differences between unstimulated primary T cells and primary T cells stimulated with αCD3 and αCD28 antibodies

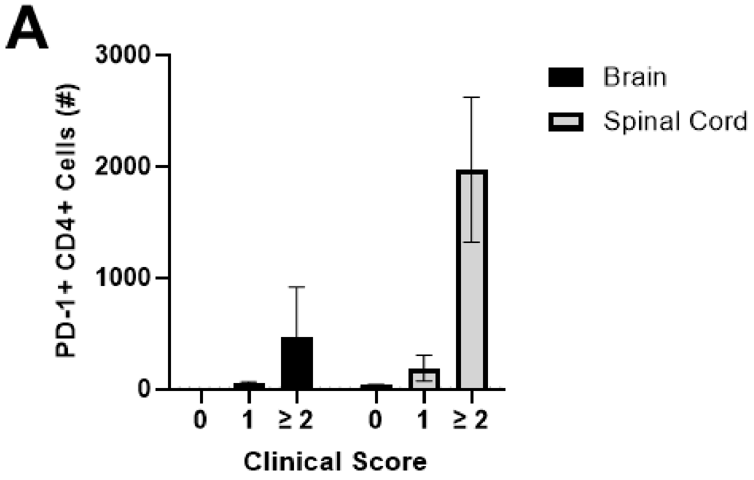

Figure S4. Number of PD-1^{+} CD4 T cells in brains and spinal cords of untreated EAE mice at different clinical scores. A) EAE was induced in 10 week-old mice using $MOG_{35\text{-}55}$, emulsified in CFA + pertussis toxin; mice were euthanized at different clinical scores and the brains and spinal cords were collected for flow cytometry analysis; at clinical scores equal or greater than 2, there are more PD-1^{+} CD4 T cells found in the respective tissues than at lower clinical scores.

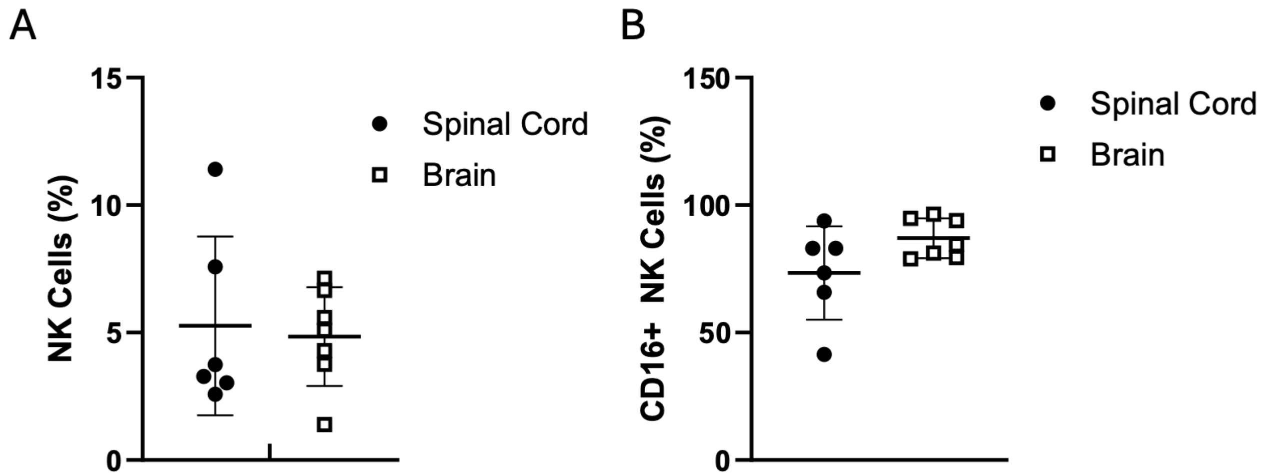

Figure S5. Percentages of NK cells found in the CNS tissue of EAE mice. (A) Fraction of NK cells among total CD45+ cells found in the brains and spinal cords of EAE mice at clinical scores greater than or equal to 2. (B) Fractions of NK cells that are positive for CD16 in the brains and spinal cords of EAE mice at clinical scores greater than or equal to 2.
